# Complement C5 Contributes to Brain Injury After Subarachnoid Hemorrhage

**DOI:** 10.1007/s12975-019-00757-0

**Published:** 2019-12-06

**Authors:** Bart J. van Dijk, Joost C.M. Meijers, Anne T. Kloek, Veronique L. Knaup, Gabriel J.E. Rinkel, B. Paul Morgan, Marije J. van der Kamp, Koji Osuka, Eleonora Aronica, Ynte M. Ruigrok, Diederik van de Beek, Matthijs Brouwer, Marcela Pekna, Elly M. Hol, Mervyn D.I. Vergouwen

**Affiliations:** 1 UMC Utrecht Brain Center, Department of Translational Neurosciences, University Medical Center Utrecht, Utrecht University, Heidelberglaan 100, Utrecht, The Netherlands; 2 UMC Utrecht Brain Center, Department of Neurology and Neurosurgery, University Medical Center Utrecht, Utrecht University, Heidelberglaan 100, Utrecht, The Netherlands; 3 Department of Experimental Vascular Medicine, Academic Medical Center, Meibergdreef 9, Amsterdam, The Netherlands; 4 Department of Plasma Proteins, Sanquin Research, Plesmanlaan 125, Amsterdam, The Netherlands; 5 Department of Neurology, Amsterdam Neuroscience, Academic Medical Center, Meibergdreef 9, Amsterdam, The Netherlands; 6 Systems Immunity Research Institute, Cardiff University, Heath Park, Cardiff, UK; 7 Department of Neurological Surgery, Aichi Medical University, 1-1 Karimatayazako, Aichi, Japan; 8 Department of Neuropathology, Academic Medical Center, Meibergdreef 9, Amsterdam, The Netherlands; 9 Department of Clinical Neuroscience, Institute of Neuroscience and Physiology, Sahlgrenska Academy at University of Gothenburg, Medicinaregatan 9A, Gothenburg, Sweden; 10 Netherlands Institute for Neuroscience, Institute of the Royal Netherlands Academy of Arts and Sciences, Meibergdreef 47, Amsterdam, The Netherlands

**Keywords:** Aneurysmal subarachnoid hemorrhage, Complement system, Brain injury

## Abstract

Previous studies showed that complement activation is associated with poor functional outcome after aneurysmal subarachnoid hemorrhage (SAH). We investigated whether complement activation is underlying brain injury after aneurysmal SAH (*n* = 7) and if it is an appropriate treatment target. We investigated complement expression in brain tissue of aneurysmal SAH patients (*n* = 930) and studied the role of common genetic variants in C3 and C5 genes in outcome. We analyzed plasma levels (*n* = 229) to identify the functionality of a single nucleotide polymorphism (SNP) associated with outcome. The time course of C5a levels was measured in plasma (*n* = 31) and CSF (*n* = 10). In an SAH mouse model, we studied the extent of microglia activation and cell death in wild-type mice, mice lacking the C5a receptor, and in mice treated with C5-specific antibodies (*n* = 15 per group). Brain sections from aneurysmal SAH patients showed increased presence of complement components C1q and C3/C3b/iC3B compared to controls. The complement component 5 (*C5*) SNP correlated with C5a plasma levels and poor disease outcome. Serial measurements in CSF revealed that C5a was > 1400-fold increased 1 day after aneurysmal SAH and then gradually decreased. C5a in plasma was 2-fold increased at days 3–10 after aneurysmal SAH. In the SAH mouse model, we observed a ≈ 40% reduction in both microglia activation and cell death in mice lacking the C5a receptor, and in mice treated with C5-specific antibodies. These data show that C5 contributes to brain injury after experimental SAH, and support further study of C5-specific antibodies as novel treatment option to reduce brain injury and improve prognosis after aneurysmal SAH.

**Electronic supplementary material:**

The online version of this article (10.1007/s12975-019-00757-0) contains supplementary material, which is available to authorized users.

## Introduction

Aneurysmal SAH is a devastating subtype of stroke, caused by rupture of an aneurysm of an intracranial artery in the subarachnoid space. Although the prognosis after aneurysmal SAH has improved over the last decades, 90-day case fatality is still around 30% in hospital-based studies [1]. The most important determinant of poor functional outcome after aneurysmal SAH is early brain injury directly related to the initial bleeding [1, 2]. Other major determinants of poor functional outcome are rebleeding of the aneurysm and delayed cerebral ischemia, which may occur 4–14 days after the initial hemorrhage [1, 3]. No treatment exists for early brain injury, while the effect of calcium antagonist nimodipine in preventing delayed cerebral ischemia is only modest [4]. Therefore, new treatment options are needed to reduce brain injury after SAH.

Poor functional outcome after SAH can partially be predicted using models that include factors such as age, World Federation of Neurological Surgeons (WFNS) scale at admission, and premorbid history of hypertension [5]. However, these models do not take into account the inflammatory response after SAH, which is considered to play a key role in the pathogenesis of early brain injury and delayed cerebral ischemia after aneurysmal SAH [6, 7]. The inflammatory response after SAH is independently associated with poor clinical condition on admission, delayed cerebral ischemia, disability, and death [6, 8, 9]. The inflammatory response in the brain is reflected by the activation of microglia and astrocytes [10]. The complement system may be a major component of this acute injury induced neuroinflammation after aneurysmal SAH. The classical pathway of the complement system is initiated by C1q, which then activates a cascade of other soluble or membrane bound proteins. Activation of the complement system leads to the cleavage of C5, resulting in C5a and the lytic C5b-9 membrane attack complex. Anaphylatoxins C3a and C5a are important proinflammatory mediators and have the potential to produce vasoconstriction and activate coagulation by aggregation of platelets and regulation of tissue factor activity [11–15]. All these processes occur after aneurysmal SAH and are associated with early brain injury and delayed cerebral ischemia [16–21]. Plasma levels of mannose-binding lectin, C3a, and C5a early after SAH correlate with outcome at discharge [22, 23]. Despite the observed associations between complement activation and measures of brain injury after SAH, it remains unclear if these are causal relationships. If so, this would represent an appealing target to decrease brain injury and improve prognosis after SAH.

We investigated the role of complement activation after SAH. In an autopsy study, we examined local complement expression in brains of patients who died from aneurysmal SAH. We evaluated the association between common complement component polymorphisms and functional outcome and delayed cerebral ischemia, and elucidated the time-course of complement activation in CSF and plasma after SAH using serial measurements. We then confirmed our results in a SAH mouse model and studied whether treatment with a C5-specific monoclonal antibody affects brain injury after SAH.

## Methods

### Autopsy Study

We used autopsy material from seven SAH patients (two males, five females, median age 56 years (range 34–77 years)) who died within 11 days after aneurysmal SAH. All patients had delayed cerebral ischemia [3]. We isolated cortical areas with both white and gray matter. From autopsy material from five control patients (three males, two females, median age 72 years (range 52–75 years)) who died from non-neurological causes (myocardial infarction: *n* = 3; heart failure: *n* = 1; pulmonary embolism: *n* = 1), we isolated corresponding areas. Sections were stained with hematoxylin and eosin to distinguish infarcted from non-infarcted areas. For analysis, only non-infarcted areas were used. Complement component C1q acts as the initiating molecule in the classical pathway of the complement cascade, whereas C3b and iC3b are biologically active fragments of C3, a central molecule in the complement system. Deposition of C3/C3b/iC3b in the tissue indicates that the complement pathway reached a proinflammatory state [24]. We performed immunohistochemistry to investigate the presence of C1q (polyclonal rabbit, anti-human C1q complement, #F0254, DAKO, 1:200) and C3/C3b/iC3b (polyclonal rabbit, anti-human C3c complement, #F0201, DAKO, 1:100). We were unable to evaluate the presence of C5a or C5b-9 (Membrane Attack Complex), as these antibodies do not work on the paraffin-embedded postmortem material. The immunohistochemistry of C1q and C3/C3b/iC3b on human brain sections resulted in heterogenous staining patterns, as shown in high magnification (× 100) images in Supplementary Fig. 1. Optical density measurements were used for quantitative assessments. Images were analyzed with FIJI software (ImageJ 2.0.0). After manually selecting the entire non-infarcted area per brain slide, selections were automatically quantified, expressed as a mean total of -log transformed grayscales, to calculate a mean per patient. The investigators who analyzed the immunohistochemistry images were blinded to the experimental group.

### Genotyping Study

The cohort of the genetic study consisted of 930 patients who were admitted between 1983 and 2011 to the University Medical Center Utrecht, the Netherlands, which is a tertiary referral center for patients with SAH (Table 1). We used the University Medical Center Utrecht Subarachnoid Hemorrhage database, which is a prospectively collected database of consecutive patients with confirmed SAH, to collect the following variables: age, sex, clinical condition on admission according to the WFNS grading scale [25], aneurysm location, the occurrence of rebleeding and delayed cerebral ischemia, and functional outcome. After centrifugation of blood samples, the cell pellets were used to extract DNA for genotyping. The primary outcome was poor functional outcome, which was defined as a Glasgow Outcome Scale score of 1–3, 3 months after ictus [26]. Secondary outcome was clinical deterioration due to delayed cerebral ischemia, for which we used the definition that was proposed by an international multidisciplinary research group [3]. The occurrence of rebleeding was recorded between admission and aneurysm treatment, and defined as a sudden clinical deterioration with signs of increased hemorrhage on CT scan compared with previous CT imaging or found at autopsy, or a sudden clinical deterioration suspect for rebleeding with fresh blood in the ventricular drain in which no CT scan or autopsy was obtained. During collection of clinical data, the investigators were blinded for the results of genotyping.

**Table 1:** Patient characteristics

	Cohort (*N* = 930)
Median age (year, interquartile range)	50 (43–59)
Female sex (number, %)	653 (70)
WFNS grading scale on admission (number/total, %)	
1 2 3 4 5	481/905 (53) 188/905 (21) 60/905 (7) 115/905 (13) 61/905 (7)
Location of aneurysm in anterior circulation (number/total, %)	802/911 (88)
In-hospital complications (number/total, %)	
Rebleeding Clinical deterioration due to delayed cerebral ischemia	125/914 (14) 174/913 (19)
Glasgow Outcome Scale Score at 3 months (%)	
1. Death 2. Vegetative state 3. Severe disability 4. Moderate disability 5. Good recovery	73 (8) 4 (0) 112 (12) 224 (24) 517 (56)

### Genotyping

The following common allele variants with a frequency of > 5% were investigated: C3 rs1047286, C3 rs2230199, and C5 rs17611. Genotyping was done using TaqMan SNP Genotyping Assays with the Lightcycler® 480. In case of unsuccessful genotyping of C5 rs17611, we used data from a previous genome-wide association study, which partly included the same patients as in the present study [27]. Due to the supplementary genome-wide association study data, genotype success rate for the C5 rs17611 SNP increased from 94.1 to 98.0%. Genotyping was performed on coded DNA samples, so clinical information remained unknown to the laboratory personnel.

### C5a Levels in Plasma of Genotyped Patients

To determine the relationship between the C5 rs17611 SNP and C5a levels in plasma, we used plasma samples in a subset of 229 patients who presented with aneurysmal SAH between 2007 and 2011 (63 males, 176 females, median age 57 years (range 19–88 years)). Blood samples were obtained in EDTA tubes between days 1 and 14 after ictus. The samples were centrifuged and C5a levels in plasma were measured with the use of Human Complement C5a ELISA Kit (LifeSpan Biosciences) according to the manufacturers’ instructions.

### Serial Measurement of C5a in CSF and Plasma

We performed serial blood withdrawals in 31 patients with aneurysmal SAH (13 males, 18 females, median age 53 years (range 32–74 years)) and single measurements in 17 healthy control patients (6 males, 11 females, median age 53 years (range 30–63 years)). Blood samples in SAH patients were obtained in citrate tubes on days 1, 3, 5, 7, 10, 14, and 17 (**±** 1 day), with day of ictus defined as day 0. The samples were centrifuged and plasma C5a levels were measured with the use of a C5a EIA kit (Quidel) according to the manufacturers’ guidelines (standard curves *R*^2^ ranging from 0.9998 to 1, lower limit of detection 0.05 ng/mL).

CSF samples were collected from 10 patients with aneurysmal SAH (three males, seven females, median age 57 years (range 41–75 years)) and three controls in whom CSF was collected during surgery for an unruptured aneurysm (one male, two females, ages 49, 60, and 60 years). In 6 patients, CSF samples were collected from external ventricular drains and in 4 patients from lumbar drains. CSF was sampled on days 1, 3, 5, 7, 10, 12, and 14 after SAH with day of ictus defined as day 0. All CSF samples were immediately centrifuged upon collection, and the supernatants were stored at − 80 °C until analysis. C5a levels were measured with the use of a C5a EIA kit (Quidel) according to the manufacturer’s guidelines.

### SAH Animal Model

To model SAH in mice, we applied the prechiasmatic blood injection model as described previously, with injection of 60 μL of blood in the prechiasmatic cistern [28, 29]. Body temperature was maintained at 37 °C. Cerebral blood flow was measured between 7.5 min prior to and up to 15 min after blood injection, with a laser Doppler flow meter (BLF22; Transonics Systems, New York, NY, USA). The success of SAH creation was confirmed by a sharp reduction in cerebral blood flow during blood injection. Mean cerebral blood flow during blood injection dropped to ≤ 25% of baseline in all groups, which is a reflection of an acute increase in intracranial pressure that is also seen in patients with aneurysmal SAH. Mice were killed 48 h after blood injection. After intracardiac perfusion-fixation with 4% paraformaldehyde in PBS, brains were removed and post-fixed for 48 h in 4% paraformaldehyde in PBS, pH 7.4. Coronal cuts were made with a mouse brain matrix (Zivic Instruments, Pittsburgh, PA, USA). Slices were dehydrated and embedded in paraffin, and cut into 7-μm sections with a microtome.

The following experimental groups were investigated: (a) wild-type (WT) mice (BALB/c, male, *n* = 15) with prechiasmatic injection of 60 μL of blood from a donor WT mouse (BALB/c, male, *n* = 15); (b) WT mice (BALB/c, male, *n* = 15) with prechiasmatic injection of 60 μL of blood from a donor WT mouse (BALB/c, male, *n* = 15) and with a subsequent intraperitoneal injection of a neutralizing monoclonal antibody directed against murine C5 (20 min after creation of SAH, 1 mg per mouse; clone BB5.1; [30]; and (c) *C5aR*^*−/−*^ mice (C.129S4(B6)-*C5ar1*^*tm1Cge*^/J, 15 times back crossed to BALB/c, obtained from the Jackson Laboratory, male, *n* = 15) with prechiasmatic injection of 60 μL of blood from a donor *C5aR*^−/−^ mouse, male, *n* = 15). All mice were 2 months of age. The experiments were performed in random order.

### Immunocytochemistry

Iba1 and cleaved caspase-3 immunofluorescence imaging was performed on coronal 7-μm sections that were taken 3 mm anterior to the cerebellum of the mice. Iba1 (polyclonal rabbit, 1:4000, #019-19741, Wako), cleaved caspase 3 (polyclonal rabbit, Antibody #9661, 1:100, Cell Signalling), and NeuN (monoclonal, 1:500, Mab377, Chemicon) were used as primary antibodies. Hoechst 33258 (1:1000, Sigma-Aldrich) was used to visualize cell nuclei. We selected four predefined areas of cerebral cortex to quantify protein expression. Images were taken with the use of an epifluorescence microscope (× 20 objective, Axio Scope A1, Zeiss) and processed with the use of Axiovision (Zeiss). We calculated the threshold area percentage of Iba1-positive cells with the use of FIJI software (ImageJ, NIH). The investigators who analyzed Iba1 and cleaved caspase 3-positive cells were blinded to the experimental group.

### Statistical Analysis—Autopsy Study

Optical density values were presented as mean with SEM, compared between areas of SAH patients and controls, and analyzed with a Student’s *t* test for the C1q analysis and a Mann-Whitney test for the C3/C3b/iC3b analysis.

### Statistical Analysis—Genetic Analysis

The number of patients in our cohort (*n* = 930) was based on a variant with a minor allele frequency of 0.36 and a study power of > 80%, to detect an association of the variant with poor functional outcome with an odds ratio of ≥ 1.8 (http://pngu.mgh.harvard.edu/~purcell/gpc/). We calculated whether the genotype frequencies concurred with the Hardy-Weinberg equilibrium by use of an *X*^2^ test with one degree of freedom with a *p* value of less than 0.05 to indicate significance. Differences in genotype frequencies were analyzed with a two-tailed *X*^2^ test. Statistical analyses were performed with SPSS version 20.0 for Windows (IBM, Armonk, NY, USA). We calculated odds ratios with 95% confidence intervals and performed logistic regression analyses with adjustments for age, sex, and WFNS grading scale on admission to calculate adjusted odds ratio. C5a levels per genotype of the C5 SNP rs17611 were analyzed by the Kruskal-Wallis test followed by Dunn’s multiple comparison test. Correlation between plasma C5a levels and functional outcome 3 months after ictus, measured with the Glasgow Outcome Scale, was analyzed by partial Spearman’s rho correlation test and controlled for WFNS grading scale score on admission.

### Statistical Analysis—Serial C5a Measurements in CSF and Plasma

Concentrations were presented as mean with SEM. Means of each serial C5a measurement in plasma were compared with the mean of the controls, calculated with ANOVA followed by the Dunnett’s multiple comparisons test. Each serial C5a measurement in CSF was compared with the controls, calculated with Kruskal-Wallis test, followed by the Dunn’s multiple comparisons test.

### Statistical Analysis—Animal Experiments

The number of mice (*n* = 15) was based on an assumed mortality rate of 6% (leaving 14 mice available for analysis), a minimum difference in extent of microglia/macrophage activation or cells undergoing apoptosis of 25% between groups with and without C5a ablation, a standard deviation of 20% in both groups, 5% error, and 80% power.

The results of Iba1 and cleaved caspase-3 immunofluorescence stainings were presented as means with SD. Differences between groups were calculated with Kruskal-Wallis test, followed by the Dunn’s multiple comparisons test. Probability values of < 0.05 were considered to be of statistical significance.

## Results

### Complement Expression in Brains of SAH Patients

**Fig. 1:**
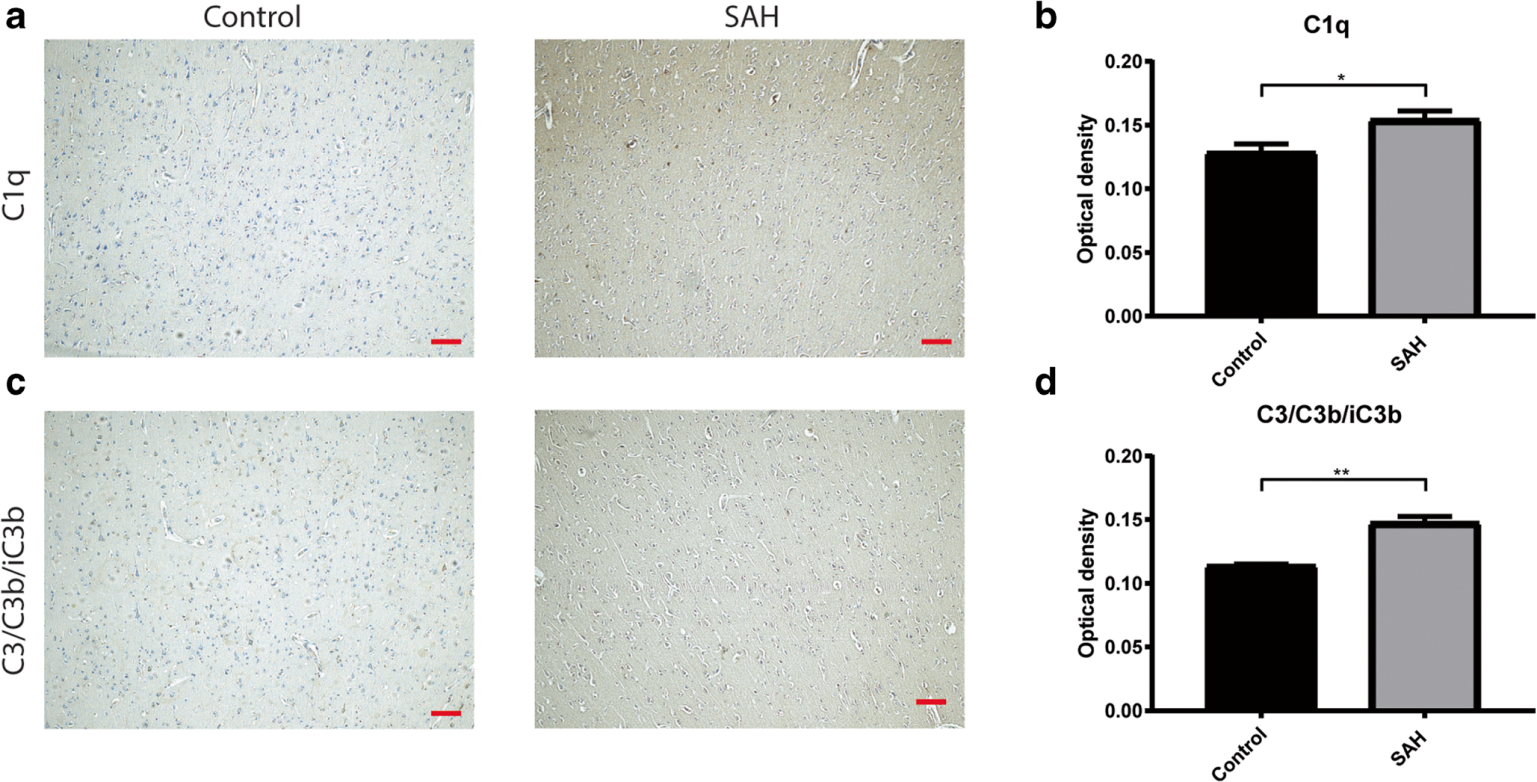
Complement expression in human autopsy brain sections. **a** Representative images of immunohistochemical staining of C1q on autopsy brain sections of a control patient, and a subarachnoid hemorrhage patient. **b** Average optical density measurements of C1q. **c** Representative images of immunohistochemical staining of C3/C3b/iC3b on autopsy brain sections of a control patient, and a subarachnoid hemorrhage patient. **d** Average optical density measurements of C3/C3b/iC3b. Scale bar: 100 μm, Student’s *t* test; *p ≤ 0.05; **p ≤ 0.01; mean ± SEM

In autopsy brain tissue from SAH patients and controls, we found a higher expression in SAH patients compared to controls of complement component C1q (control mean ± SEM 0.129 ± 0.007, *n* = 5, versus SAH mean ± SEM 0.155 ± 0.007, *n* = 7; *t* = 2.66, *df* = 10; *p* < 0.05, Fig. 1a and b) and complement component C3/C3b/iC3b (control mean ± SEM 0.114 ± 0.001 versus SAH mean ± SEM 0.148 ± 0.005; *U* = 0, *p* < 0.01, Fig. 1c and d). These results show that C1q and C3/C3b/iC3b immunoreactivity is increased in the brain after SAH.

### Genetic Association Study of Common Allele Variants of the Complement C3 and C5 Genes

Inter-individual differences in the inflammatory response resulting from common polymorphisms in the complement system may explain part of the heterogeneity in disease severity and outcome following aneurysmal SAH. Previously, it was shown that these polymorphisms are involved in central nervous system inflammation [31]. We performed a genetic association study to investigate if common allele variants in C3 and C5 genes are associated with functional outcome and delayed cerebral ischemia. Characteristics of the 930 patients included in the genetic study are shown in Table 1. Poor functional outcome, measured by the Glasgow Outcome Score 1–3 at 3 months, occurred in 189 of the 930 patients (20%). Data on genotyping success rate are shown in Table 2. All genotype frequencies were in concurrence with the Hardy-Weinberg equilibrium.

**Table 2:** Allele frequency, Hardy-Weinberg equilibrium, and genotyping success rate of common complement component polymorphisms in 930 patients with aneurysmal subarachnoid hemorrhage

Gene	SNP ID	A %	B %	AA	AB	BB	HWE *p* value	Success rate
C3	rs1047286	80.1%	19.9%	545	267	35	*p* = 0.750	91%
C3	rs2230199	77.6%	22.4%	590	277	37	*p* = 0.534	97%
C5	rs17611	43.7%	56.3%	182	432	297	*p* = 0.274	98%

*HWE* Hardy-Weinberg equilibrium, *SNP* single nucleotide polymorphism

The relationship between genotyping and outcome is shown in Table 3. The C3 SNP frequencies were similar in patients with poor and good functional outcome, and in patients with and without delayed cerebral ischemia. The C5 rs17611 allele A was associated with poor functional outcome (odds ratio 1.49; 95% confidence interval 1.04–2.14; adjusted odds ratio 1.53; 95% CI 1.02–2.28; Table 3), but not with delayed cerebral ischemia (odds ratio 1.09; 95% CI 0.78–1.52; adjusted odds ratio 1.10; 95% confidence interval 0.79–1.55; Table 4).

**Table 3:** Genotyping analysis of common complement component polymorphisms in 741 patients with favorable outcome and 189 patients with unfavorable outcome

Gene	Allele	SNP ID	Favorable outcome			Unfavorable outcome			OR (95%CI)	aOR (95%CI)
			AA	AB	BB	AA	AB	BB		
C3	**C**/T	rs1047286	422	220	27	123	47	8	1.31 (0.92–1.87)	1.46 (0.98–2.17)
C3	**C**/G	rs2230199	462	227	28	128	50	9	0.84 (0.59–1.18)	0.75 (0.51–1.11)
C5	**A**/G	rs17611	144	333	249	38	99	48	1.49 (1.04–2.14)	1.53 (1.02–2.28)

Allele in bold is used for analysis

*aOR* adjusted odds ratio (adjusted for gender, age at time of ictus, and WFNS grading scale on admission), *CI* confidence interval, *OR* odds ratio, *SNP* single nucleotide polymorphism

**Table 4:** Genotyping analysis of common complement component polymorphisms in 725 patients without DCI and 169 patients with DCI

Gene	Allele	SNP ID	No DCI			DCI			OR (95%CI)	aOR (95%CI)
			AA	AB	BB	AA	AB	BB		
C3	**C**/T	rs1047286	432	214	28	103	47	7	0.93 (0.67–1.30)	0.95 (0.67–1.33)
C3	**C**/G	rs223019	467	222	29	113	49	7	1.11 (0.80–1.53)	1.10 (0.79–1.53)
C5	**A**/G	rs17611	152	334	239	27	91	51	1.09 (0.78–1.52)	1.10 (0.79–1.55)

Allele in bold is used for analysis

*aOR* adjusted odds ratio (adjusted for gender, age at time of ictus, and WFNS grading scale on admission), *CI* confidence interval, *DCI* delayed cerebral ischemia, *OR* odds ratio, *SNP* single nucleotide polymorphism

**Fig. 2:**
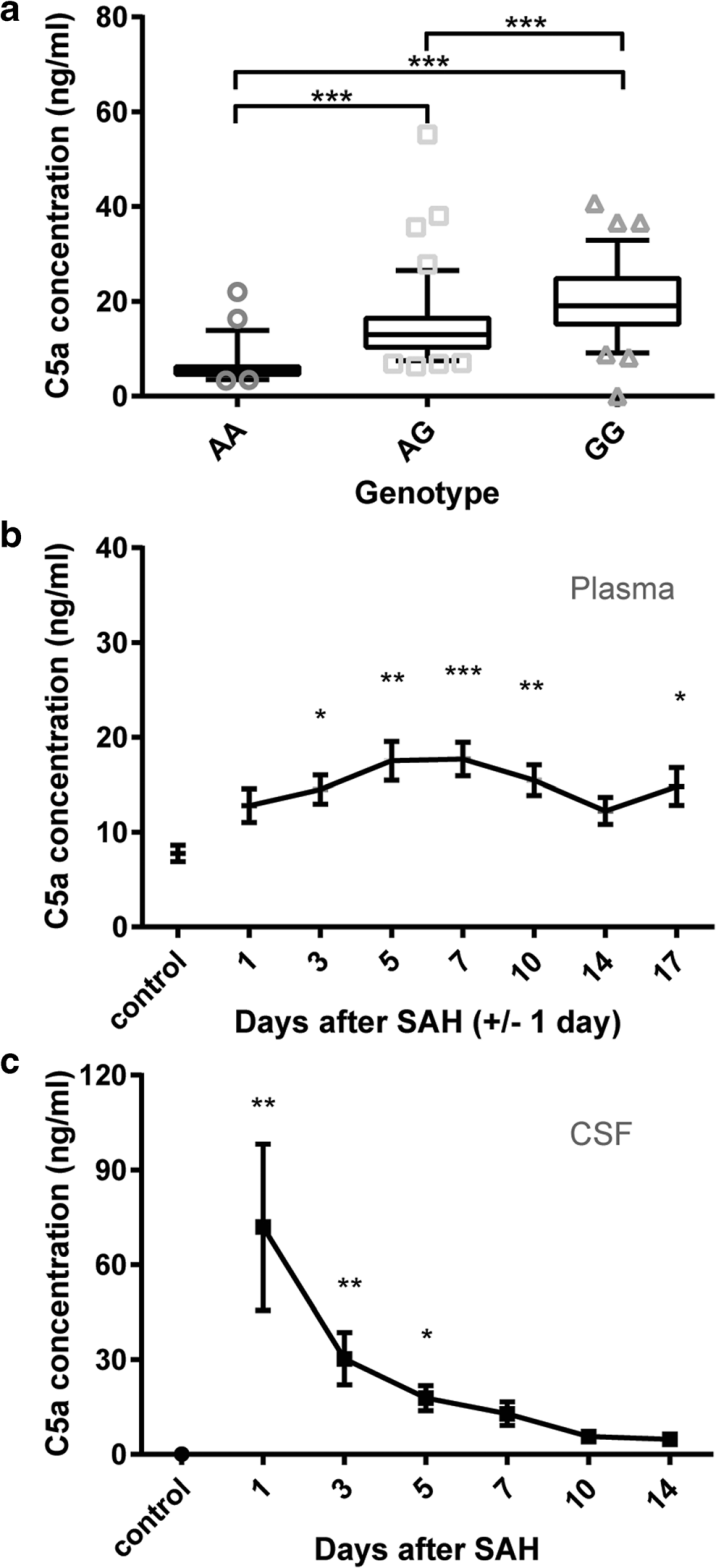
C5a levels measured in plasma and CSF of aneurysmal subarachnoid hemorrhage patients. **a** Plasma C5a levels (ng/mL) of genotyped patients, grouped per genotype of the C5 single nucleotide polymorphism rs17611; blood samples were obtained between day 1 and day 14 postsubarachnoid hemorrhage; AA; *n* = 53, AG; *n* = 98, GG; *n* = 78; Kruskal-Wallis, Dunn’s multiple comparison, ***p ≤ 0.001, median ± 5–95 percentile. **b** Sequentially measured C5a levels in plasma of aneurysmal subarachnoid hemorrhage patients (*n* = 31), versus plasma C5a levels of healthy controls (*n* = 17). Blood samples were taken on days 1, 3, 5, 7, 10, 14, and 17 (± 1 day) after subarachnoid hemorrhage. *p ≤ 0.05, **p ≤ 0.01, ***p ≤ 0.001, ANOVA, Dunnett’s post hoc; mean ± SEM. **c** Sequentially measured C5a levels in CSF of aneurysmal subarachnoid hemorrhage patients (*n* = 10) versus CSF C5a levels of patients with an unruptured aneurysm (*n* = 3). CSF obtained on days 1, 3, 5, 7, 10, 12, and 14 after subarachnoid hemorrhage. *p ≤ 0.05, **p ≤ 0.01, Kruskal-Wallis test, Dunn’s post hoc; mean ± SEM

Since the C5 rs17611 allele A was associated with poor functional outcome, we subsequently measured plasma C5a levels in 229 genotyped patients with SAH in blood samples drawn between days 1 and 14 after the hemorrhage. Patients carrying allele A of the C5 rs17611 SNP had lower plasma C5a levels (genotype AA median 5.3 ng/mL [95% confidence interval 5.3–7.1 ng/mL]; AG median 13.0 ng/mL [95% confidence interval 13.1–16.0 ng/mL]; GG median 19.3 ng/mL [95% confidence interval 18.2–25.9 ng/mL]; *H* = 117.9, *p* < 0.0001, Fig. 2a). No correlation was found between plasma C5a levels and poor functional outcome, measured by the Glasgow Outcome Scale, 3 months after subarachnoid hemorrhage (partial Spearman’s rho correlation − 0.09, *p* = 0.16, controlled for WFNS grading scale on admission).

### Serial C5a Measurements in CSF and Plasma of Subarachnoid Hemorrhage Patients

We investigated the time-course of plasma C5a levels in 31 patients with aneurysmal SAH. Blood samples were taken at days 1, 3, 5, 7, 10, 14, and 17 (± 1 day) after aneurysmal SAH. Plasma C5a levels steadily increased with a peak on day 5 after SAH (control mean ± SEM 7.8 ± 0.9 ng/mL; SAH day 5 mean ± SEM 17.9 ± 2.0 ng/mL, *F* = 3.305, *p* < 0.01) and dropped thereafter (Fig. 2b). Furthermore, we used serial CSF samples from 10 patients with aneurysmal SAH up to day 14 after ictus and single CSF samples from 3 controls with unruptured aneurysms. The CSF levels of C5a were > 1400 times increased 1 day after aneurysmal SAH (control mean ± SEM 0.05 ± 0.03 ng/mL; SAH day 1 mean ± SEM 71.9 ± 26.4 ng/mL, *H* = 23.21, *p* < 0.001) and slowly decreased over time (Fig. 2c). These data show that complement activation is strongly increased in the acute phase of SAH, in particular in the central nervous system.

### Functional Analysis of the Role of C5 and C5a in an SAH Mouse Model

**Fig. 3:**
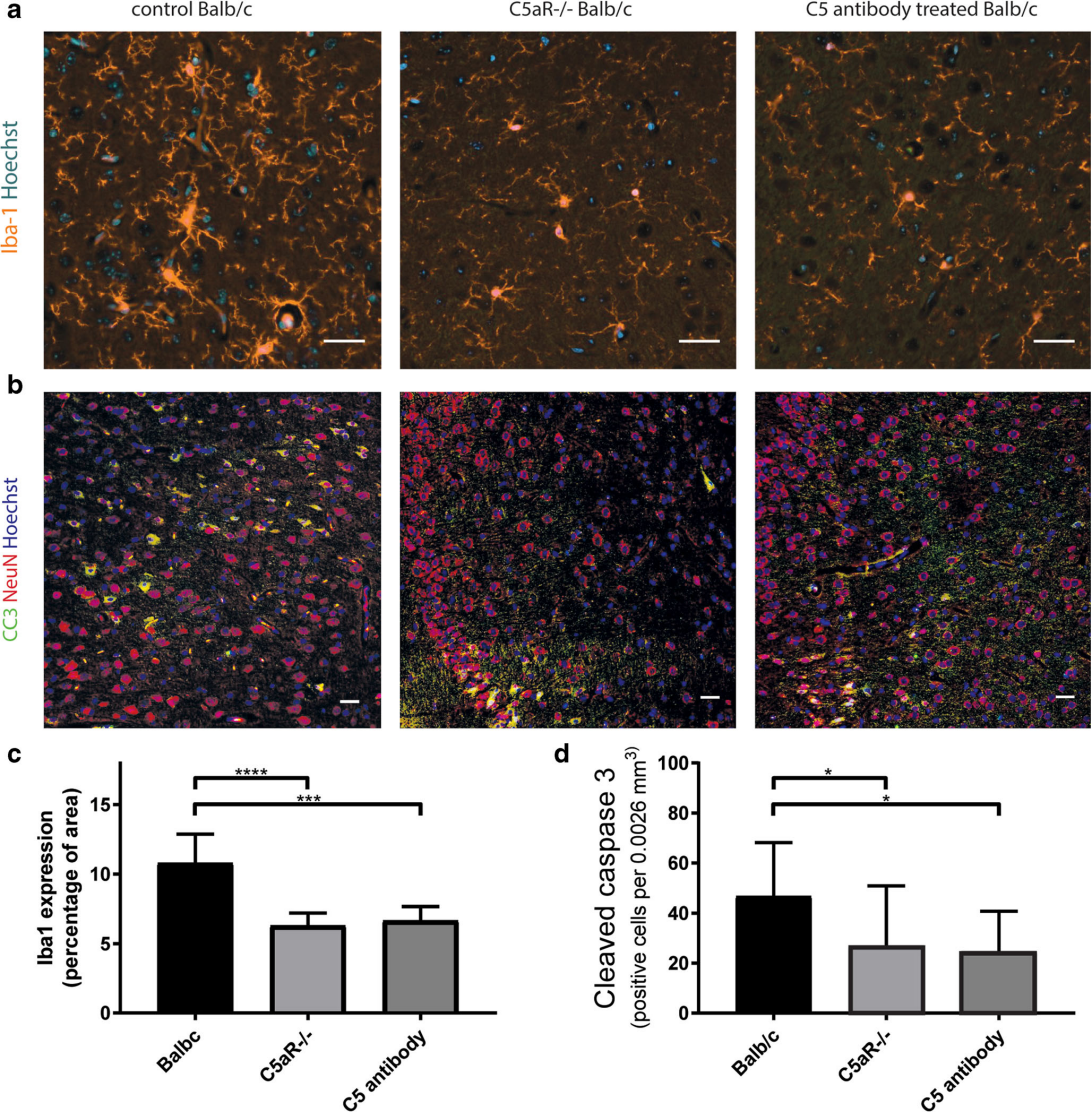
C5 antibody treatment reduces brain injury in experimental subarachnoid hemorrhage. **a** Representative images of mouse cortex stained with antibody against Iba-1 (orange); cell nuclei in blue (Hoechst). **b** Representative images of mouse cortex stained with antibody against cleaved caspase 3 (CC3; green); NeuN (red) and cell nuclei in blue (Hoechst). **c** C5a receptor deficient (C5aR^−/−^) and wild-type mice treated with C5-specific antibodies had a > 38% reduction in microglia/macrophage activation compared to untreated wild-type mice. Kruskal-Wallis, Dunn’s post hoc. **d** C5aR^−/−^ mice and control BALB/c mice treated with C5-specific monoclonal antibodies had > 39% reduction in cells undergoing apoptosis, compared to untreated wild-type mice 48 h after subarachnoid hemorrhage. Kruskal-Wallis, Dunn’s post hoc; *n* = 15 mice per group; ****p ≤ 0.0001, ***p ≤ 0.001, *p ≤ 0.05; mean ± SD; scale bars 30 μm.

To investigate if C5 contributes to brain injury after SAH and to study the effect of C5-specific antibodies on the extent of brain injury, we used 3 groups of mice in which SAH was created with the prechiasmatic blood injection model [29]. We assessed microglia/macrophage activation and cell death 48 h after SAH induction. We found that Iba1 expression, a microglia/macrophage marker, was reduced in the C5aR^−/−^ mice compared to wild-type controls. Moreover, the mice treated with C5 specific antibody showed similar reduction in Iba1 expression (C5aR^−/−^ mean ± SD 6.3 ± 0.9%; control BALB/c mean ± SD 10.8 ± 2.1%; C5 antibody treated mice mean ± SD 6.7 ± 1.0%; Kruskal-Wallis, Dunn’s post hoc, *H* = 22.9, *p* ≤ 0.001; Fig. 3a; quantified in Fig. 3c), indicating a reduced activation of microglia/macrophages, the innate immune cells of the brain. No difference in Iba1 expression between the C5aR^−/−^ and C5 antibody treated wildtype mice was found, showing the potency of this treatment. Furthermore, the density of cells positive for cleaved caspase 3, marker for cells undergoing apoptosis, was reduced to a similar degree in the C5aR^−/−^ mice and mice treated with C5 antibody compared to wild-type controls (C5aR^−/−^ mean ± SD 28 ± 24; C5 antibody treated BALB/c mean ± SD 26 ± 16; control BALB/c mean ± SD 47 ± 22; Kruskal-Wallis, Dunn’s post hoc, *H* = 8.6, *p* ≤ 0.05; Fig. 3b; quantified in Fig. 3d), indicating a reduction in the number of cells undergoing apoptosis. The majority of cleaved caspase 3-positive cells were also positive for NeuN, a neuronal marker (Fig. 3b).

## Discussion

We are the first to show in a multilevel approach that complement factor C5 contributes to brain injury after SAH. We showed that the complement system is activated in the brain following SAH, as reflected by the increased immunoreactivity for C1q and C3/C3b/iC3b in brain tissue of patients who died after SAH compared with brain tissue of patients who died from non-neurological causes. C5 rs17611 SNP correlated with functional outcome after SAH and with plasma C5a levels, and that C5a levels in CSF were markedly increased on day 1 after ictus and gradually decreased within the next 2 weeks. Plasma C5a levels were increased at a later stage, with a peak concentration around days 3–10 after ictus. In a mouse model of SAH, mice lacking C5a receptor had a ≈ 40% reduction of brain injury as reflected by reduced microglia/macrophage activation and cell apoptosis. This indicates that C5a is involved in the pathogenesis of brain injury after experimental SAH. Similarly, mice treated with C5 neutralizing antibodies directly after the hemorrhage had reduced brain injury. As the mice were treated short after the induction of SAH and sacrificed 2 days after SAH, this suggests that the inhibition of C5 has a long-lasting effect. These data indicate that C5 antibodies may be a promising new treatment option to decrease brain injury. Importantly, C5 antibodies are already used for other clinical indications [32–34]. Furthermore, studies showed that C5 antibodies are a promising new treatment option for patients with other neurological diseases, such as pneumococcal meningitis, neuromyelitis optica, and myasthenia gravis [31, 35, 36].

The C5 rs17611 SNP correlated with poor functional outcome. This SNP has also been linked to poor outcome after pneumococcal meningitis [31], adverse cardiovascular outcomes [37], and periodontitis [38]. Recently, the functional basis for these disease associations was clarified [39]. Allele G of the C5 rs17611 SNP causes a missense mutation in the C5 gene, increases the rate of proteolytic activation of C5 and C5a generation. As a result, patients carrying allele G have increased C5a plasma levels [39]. This is in accordance with the results from our study, in which we found that patients carrying allele G of the C5 rs17611 SNP had higher plasma C5a levels than patients with allele A. Although patients with allele A had lower plasma C5a levels and were at increased risk of poor functional outcome, the plasma levels were not correlated with poor functional outcome. Since complement activation was much stronger upregulated in the CSF than in plasma, it remains to be investigated if C5a levels in CSF correlate better with functional outcome than C5a levels in plasma.

There are a number of mechanisms by which complement activation can contribute to brain injury. The complement system has been linked to the regulation of synapse numbers [40]. In particular, complement components C1q and C3 have been implicated to facilitate the removal of synapses [41]. Unwanted synapses are tagged with C1q and C3, thereby becoming eligible for elimination. Microglia recognize these components and start to remove the synapse [42]. Synaptic pruning by microglia occurs during development, but also in diseases such as glaucoma and Alzheimer’s disease and after viral infection [41, 43, 44]. Therefore, complement activation in response to SAH may also induce excessive synapse pruning. However, while early complement components are involved in synapse elimination, the involvement of downstream components, such as C5 and C5a, remain to be investigated. Another mechanism by which complement activation may contribute to brain injury is by the membrane attack complex, formed by C5b-C9 complexes. Hemolysis of blood in the CSF is thought to be complement system mediated by activation of the membrane attack complex [45, 46]. The membrane attack complex may also bind bystander cells, such as endothelial cells, ependymal cells and other brain cells, and thereby induce brain injury.

To address the specific involvement of C5 and C5a in brain injury after SAH, we used *C5aR*^*−/−*^ and wild-type mice treated with neutralizing antibody against C5. While the antibody treatment inhibits the generation of both activation products of C5, namely C5a and C5b, only the C5aR-mediated functions of C5a are absent in the *C5aR*^*−/−*^ mice. Our experimental data show that apoptosis and microglia activation are reduced in mice lacking C5aR as well as mice treated with a single dose of neutralizing antibody against C5. These findings provide evidence for the detrimental role of C5a in brain injury after SAH and are in line with previous studies in which C5aR deficiency as well as treatment with a C5aR antagonist resulted in reduced apoptosis, increased cell viability, and reduced infarct volume after ischemic stroke [47, 48]. As C5a promotes neuronal apoptosis by acting directly through neuronal C5aR in-vitro [48], it is conceivable that the same neuronal mechanism is involved in brain injury after SAH.

C5aR is also expressed by microglia which upregulate C5aR in response to injury [49–51]. Thus, the reduced activation of microglia in the *C5aR*^*−/−*^ and anti-C5 antibody treated mice may not only be an indirect effect of reduced cell death but may also be explained by direct effects of the intervention on these cells. In experimental spinal cord injury, C5aR signaling in the acute phase has been shown to contribute to tissue damage through local proinflammatory cytokine production and the recruitment of inflammatory monocytes/macrophages [52]. As Iba1 is expressed in microglia as well as in blood born monocytes/macrophages, it is possible that recruited inflammatory cells together with activated microglia play a role in SAH-induced brain injury.

The effects of C5a in the context of brain tissue injury may not be solely detrimental. C5a has been shown to be neuroprotective during neuronal maturation [53] and to protect neurons against glutamate-mediated toxicity [54]. Through the upregulation of microglial glutamate receptor GLT-1, C5a can also increase the capacity of microglia to clear excessive glutamate [55]. In addition, in the postacute stage after spinal cord injury, signaling through the C5a-C5aR axis appears to serve a protective and/or reparative role [52]. Thus, in light of the potential beneficial effects of C5a, the timing of the therapeutic intervention targeting C5aR after SAH may need to be carefully determined.

Interestingly, C5a can bind to Gpr77, also termed C5a-like receptor 2 (C5L2; [56]). The function of C5L2 is controversial, as both pro- and anti-inflammatory properties of this receptor have been described [56]. Our results that C5aR deficiency and inhibition of C5 activation reduced brain injury to the same extent support the contention that the deleterious effects of C5a in the acute phase after SAH are mediated mainly by its canonical receptor C5aR. In addition to the release of C5a, the proteolytic activation of C5 triggers the formation of the membrane attack complex, C5b-9, leading to neuronal death through apoptosis [57] or cell lysis [55]. However, in light of our findings of the comparable effect of C5aR deficiency and anti-C5 antibody treatment, a substantial contribution of C5b-9 to brain injury after SAH appears unlikely.

There are potential limitations to our study. The human brain tissue used in the autopsy study was from deceased patients. The increased expression of complement C1q and C3/C3b/iC3b may not reflect the situation of patients with a more favorable outcome. However, the CSF used to measure C5 levels was of patients of both favorable and unfavorable outcomes, and showed a sharp increase in complement C5 levels after SAH. This suggests activation of the complement cascade both in good and poor grade SAH.

In conclusion, the present study highlights the role of C5a in the development of brain injury after SAH and identifies C5 antibodies as a potential novel treatment strategy to reduce brain injury after SAH.

## Electronic Supplementary Material

ESM 1 Heterogenous complement expression in human autopsy brain sections. A) Image of immunohistochemical staining of C1q on autopsy brain sections of a control patient with only little immunoreactivity; B) Image of immunohistochemical staining of C1q on autopsy brain sections of a SAH patient with high immunoreactivity; C) Image of immunohistochemical staining of C3/C3b/iC3b on autopsy brain sections of a control patient with little immunoreactivity; D) Image of immunohistochemical staining of C3/C3b/iC3b on autopsy brain sections of a SAH patient with high immunoreactivity; 100x magnification. (JPG 4618 kb)
