# Supplementary figures and images for: Complement C5 Contributes to Brain Injury After Subarachnoid Hemorrhage
Source: Transl Stroke Res. 2019 Dec 6;11(4):678–88. doi: 10.1007/s12975-019-00757-0 (PMC7340633; doi:10.1007/s12975-019-00757-0)

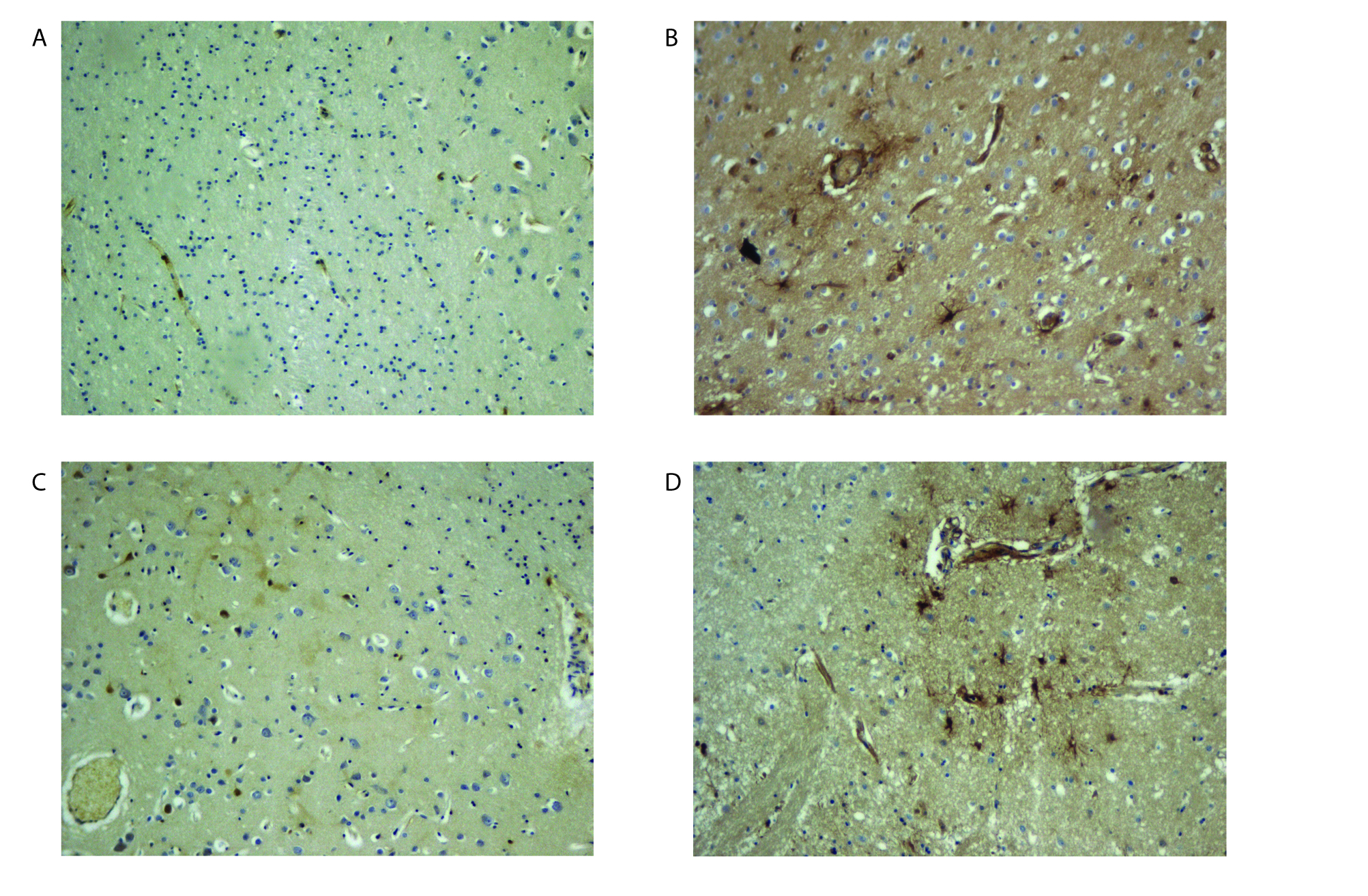

Supplement: Supplementary file 1 — Heterogenous complement expression in human autopsy brain sections. A) Image of immunohistochemical staining of C1q on autopsy brain sections of a control patient with only little immunoreactivity; B) Image of immunohistochemical staining of C1q on autopsy brain sections of a SAH patient with high immunoreactivity; C) Image of immunohistochemical staining of C3/C3b/iC3b on autopsy brain sections of a control patient with little immunoreactivity; D) Image of immunohistochemical staining of C3/C3b/iC3b on autopsy brain sections of a SAH patient with high immunoreactivity; 100x magnification. (JPG 4618 kb) [file 12975_2019_757_MOESM1_ESM.jpg]
